# Clinicians’ experiences of using and implementing a medical mobile phone app (QUiPP V2) designed to predict the risk of preterm birth and aid clinical decision making

**DOI:** 10.1186/s12911-021-01681-w

**Published:** 2021-11-18

**Authors:** N. Carlisle, H. A. Watson, J. Carter, K. Kuhrt, P. T. Seed, R. M. Tribe, J. Sandall, A. H. Shennan

**Affiliations:** Department of Women and Children’s Health, School of Life Course Sciences, King’s College London, St Thomas’ Hospital, 10th Floor North Wing, Westminster Bridge Road, London, SE1 7EH UK

**Keywords:** Preterm birth, App, QUiPP, Threatened preterm labour, Decision-making

## Abstract

**Background:**

As the vast majority of women who present in threatened preterm labour (TPTL) will not deliver early, clinicians need to balance the risks of over-medicalising the majority of women, against the potential risk of preterm delivery for those discharged home. The QUiPP app is a free, validated app which can support clinical decision-making as it produces individualised risks of delivery within relevant timeframes. Recent evidence has highlighted that clinicians would welcome a decision-support tool that accurately predicts preterm birth.

**Methods:**

Qualitative interviews were undertaken as part of the EQUIPTT study (The Evaluation of the QUiPP app for Triage and Transfer) (REC: 17/LO/1802) which aimed to evaluate the impact of the QUiPP app on management of TPTL. Individual semi-structured telephone interviews were used to explore clinicians’ (obstetricians’ and midwives’) experiences of using the QUiPP app and how it was implemented at their hospital sites. Thematic analysis was chosen to explore the meaning of the data, through a framework approach.

**Results:**

Nineteen participants from 10 hospital sites in England took part. Data analysis revealed three overarching themes which were: ‘experience of using the app’, ‘how QUiPP risk changes practice’ and ‘successfully adopting QUiPP: context is everything’. With these final themes we appeared to have achieved our aim of exploring the clinicians’ experiences of using and implementing the QUiPP app.

**Conclusion:**

This study explored different clinician’s experiences of implementing the app. The organizational and cultural context at different sites appeared to have a large impact on how well the QUiPP app was implemented. Future work needs to be undertaken to understand how best to embed the intervention within different settings. This will inform scale up of QUiPP app use across the UK and ensure that clinicians have access to this free, easy-to-use tool which can positively aid clinical decision making when caring for women in TPTL.

***Clinical trial registry and registration number*:**

ISRCTN 17846337, registered 08th January 2018, https://doi.org/10.1186/ISRCTN17846337.

## Background

It is difficult for clinicians to accurately determine which women with threatened preterm labour (TPTL) are at true risk of preterm birth. As the vast majority of women who present in TPTL will not deliver early, clinicians need to balance the risks of over-medicalising the majority of women, against the potential risk of preterm delivery for those discharged home [1]. When managing the risks of preterm birth, clinicians may prefer to ‘err on the side of caution’ [2], an attitude which appears to be reflected in the current preterm birth guidance in England [3]. However less wary clinicians are cautious of over-medicalisation [4] and as a result there are now wide variations in practice [5].

The QUiPP app is a free, validated app which can support clinical decision-making as it produces individualised risks of delivery within relevant timeframes [6–9]. The current version (Version 2) of the QUiPP predictor (for symptomatic women arriving in threatened preterm labour) is based on over 1000 women [9]. The risk score it produces is based upon the presence or absence of major risk factors, her clinical quantitative fetal fibronectin (qfFN) result and/or transvaginal ultrasound measurement of cervical length (CL). It has recently been recommended by NHS England [10], and a toolkit for implementation in hospital sites has been produced [11]

**Fig. 1:**
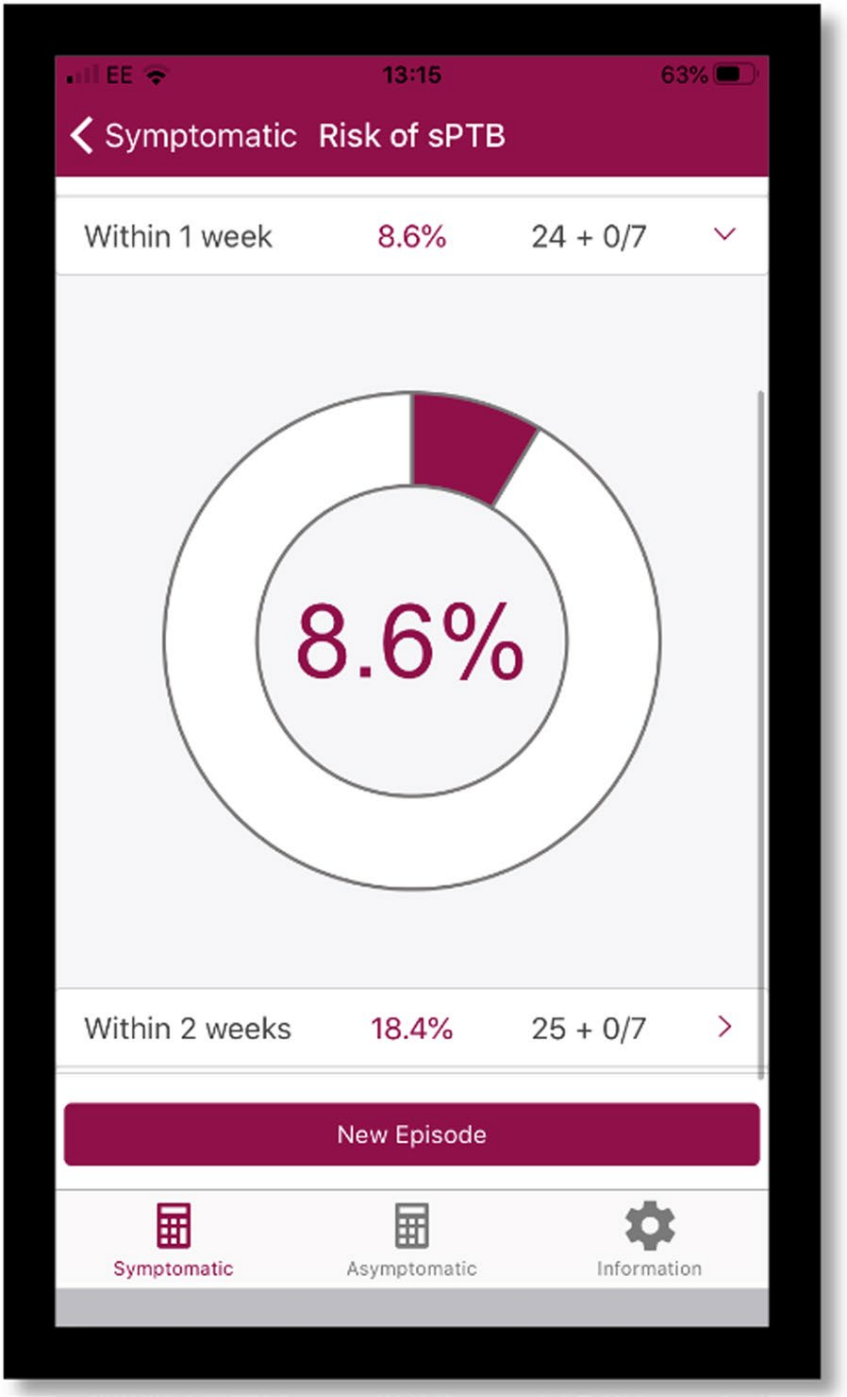
An image generated using the QUiPP app and knowledge of risk factors, cervical length and fetal fibronectin measurement. *Risk is displayed as a donut risk infographic. In this example, a woman has an 8.6% risk of delving with 1 week of the test and a 18.4% risk of delivering within 2 weeks. When clicking on the risk score, the donut risk infographic appears below. Image used with consent from the QUiPP Team

Decision support which includes a risk score with guidance, such as a cut-off for intervention, may be useful. Findings from our Delphi survey of preterm birth specialists suggested that when women arrive in TPTL, a QUiPP app score of ≥ 5% risk of delivery within a week could help guide who should be admitted [12]. The QUiPP app can also display the risk score in an infographic donut, allowing clinicians to better communicate with women who prefer visual representations of risk (Fig. 1).

There is complicated and delicate reasoning that occurs behind obstetric decision-making [13] and recent evidence has highlighted that clinicians would welcome a decision-support tool that accurately predicts preterm birth [2].

As digital technology becomes more popular in healthcare, and in maternity care [14], there are concerns that utilising this technology to triage could reduce the likelihood of patients being assessed holistically [15]. Specific qualitative research on clinicians’ decision making when caring for women with TPTL is lacking. This paper reports findings of a study that explored clinicians’ experiences of using and implementing the QUiPP app in clinical practice in England.

## Methods

### Study design

*Q*ualitative interviews were undertaken as part of the EQUIPTT study (The Evaluation of the QUiPP app for Triage and Transfer) (REC: 17/LO/1802) [16–18] which aimed to evaluate the impact of the QUiPP app on management of TPTL. The interviews were used to explore clinicians’ experiences of using the QUiPP app and how it was implemented at their hospital sites.

The study was carried out with participants from 10 of the 13 EQUIPTT study hospital sites, (geographically situated in three areas across England: London, the South East of England, and the Midlands area of England). Clinicians (midwives and obstetric doctors) were eligible if they had experience of the QUiPP app as part of TPTL management during the EQUIPTT trial. In England, while obstetricians, not midwives, make management of care decisions regarding women in TPTL, it is midwives who first assess women when they arrive to hospital. Furthermore, one hospital in this study worked under their local guidance which permitted midwives to undertake speculums and qfFN swabs on women under 37 weeks’ gestation, meaning the midwives are undertaking the predictive test required for input in the QUiPP app. This provides obvious benefits with time efficiency when triaging women, and we are aware that undertaking this change to local guidance is gaining traction with other hospitals across the United Kingdom. Midwives are therefore welcome to use the app and encouraged to. This also provides the benefit of promoting multidisciplinary decision making and cohesion which is important to promote in light of the recent Ockenden Report [19] (a report reviewing failings in maternity care in England).

The Framework approach was utilised [20], which is a systematic model of qualitative data analysis that is widely used in health research [21]. Purposive sampling was undertaken, ensuring the sample included clinicians who worked in both tertiary and secondary care settings, midwives and obstetricians, and junior and senior staff. This sample also included those who oversaw the research project at their unit with knowledge of the app but who were not using it directly in clinical care (showing or advising others to use the app rather than directly using it on women with TPTL symptoms themselves). This meant that the views of the multi-disciplinary team could be gathered, and a diversity of perspectives explored.

Potential clinicians were identified through sending an invitation by email to the principal investigators and research midwives at the 13 EQUIPTT sites who forwarded the invitation to their colleagues. Interested clinicians were given a Participant Information Leaflet and had the opportunity to ask any questions. Informed written consent was obtained before the interview was conducted via telephone at the participant’s convenience.

### Data collection

All clinician interviews were conducted between January and March 2019 via telephone and recorded with consent on encrypted digital audio equipment. As recordings were made there were no field notes. The recordings were then uploaded onto a secure, password protected University approved computer. Data collection and handling was General Data Protection Regulation (GDPR) compliant.

The semi-structured interview schedule was designed by two researchers (NC and HW) who have both clinical and research experience. This ensured it was robust enough to meet the aims of the study while ensuring clinical significance. Designing the schedule included building on previous research findings in this area and ensuring prompts/questions were included that covered the main aims and issues that this study was trying to achieve. Prompts were used to encourage expansion on certain points and encourage more detailed discussion on topics as they arose. Interview techniques aimed to build rapport [22] by active listening, summarising responses to check understanding and rephrasing of questions which were not fully answered the first time. As the participants were busy clinicians, we ensured that the interviews lasted no longer than 45 min. Most of the interviews took 20–30 min.

The interview schedule covered the background of the participant (their role, how long they had been involved in making decisions around the care of women with TPTL, and if they worked in a district general or tertiary unit), their knowledge and experience of the QUiPP app, and their opinions and values on its use.

In order to reduce researcher bias, the participants who were midwives had their interviews undertaken by a researcher who was also an obstetrician (HW), and obstetricians were interviewed by a researcher who was also a midwife (NC).

### Data analysis

The interview recordings were transcribed by a third party. Data were anonymised and given a study identification number. Two researchers (NC and HW) then checked the typed transcriptions for accuracy relative to the recordings.

Firstly, the data were indexed, and participants’ characteristics were identified (if they were a midwife or obstetrician, and if they worked in a tertiary or district general unit). Thematic analysis was chosen to explore the meaning of the data, through a framework approach [23, 24]. The contents of the transcripts were analysed by the two researchers and coded according to emergent themes. After listening to and reading the transcripts several times, a coding scheme was developed using the software NVivo 12 Pro [25]. Constant communication between the two researchers ensured they corroborated on the themes that were being produced. Utilising two researchers ensured that interpretation bias was minimised.

The coding scheme’s descriptive labels were applied to the raw data in the transcripts. This coding scheme was then progressively refined into key themes which reflected the key research questions and allowed comparisons within and between cases. In order to understand and attempt to explain the patterns between these classified experiences, a case comparative model was adopted to explore the contextual conditions which may be associated with our findings [26].

#### Reflexivity

As NC and HW are researchers and clinicians who were involved with the development and evaluation of the QUiPP app and coordination of the EQUIPTT trial, it was essential that bias was minimised when analysing the data. Reflexive sensitivity was required to ensure the researchers existing theories about the utility of the QUiPP app were not simply corroborated. Negative, unexpected and conflicting findings were actively sought, examined in detail [27] and have been highlighted in the discussion section of this manuscript. If any clinical concerns were noted during the interview these would have been raised with the individual clinicians and sites directly to avoid these clinical findings over-shadowing exploration of the interviewees’ experiences.

### Findings

Nineteen participants gave their consent and took part in individual semi-structured telephone interviews (Table 1). As highlighted in the table, some participants were not involved in direct clinical care during the trial and were responsible for research delivery (showing or advising others to use the app) at their local hospital. However, none of the participants have been involved in the development of the QUiPP app, or were part of the co-ordinating research team for the EQUIPTT trial.

**Table 1:** Table to describe the EQUIPTT clinician interview study participants

Participant number	Job title	Hospital site
1	Research midwife^a^ (a registered midwife with clinical experience who ensures research delivery and recruitment at their local hospital)	Tertiary
2	Research midwife^a^	DGH (District General Hospital)
3	Research midwife^a^	Tertiary
4	Research midwife^a^	DGH
5	Research midwife^a^	DGH
6	Consultant obstetrician^b^	DGH
7	Consultant obstetrician^b^	DGH
8	MAU (Maternity Assessment Unit or Triage) coordinating midwife^b^	DGH
9	MAU midwife^b^	DGH
10	MAU midwife^b^	DGH
11	Consultant obstetrician^b^	DGH
12	Research midwife^a^	DGH
13	Consultant obstetrician^b^	DGH
14	ST3 obstetrics^b^	DGH
15	Consultant obstetrician^b^	DGH
16	MAU midwife^b^	Tertiary
17	Research midwife^a^	DGH
18	Speciality obstetric registrar^b^	Tertiary
19	Consultant obstetrician^b^	Tertiary

^a^Not involved in direct clinical care during the trial

^b^Involved in direct clinical care during the trial

Data analysis revealed three overarching themes which were: ‘experience of using the app’, ‘how QUiPP risk changes practice’ and ‘successfully adopting QUiPP: context is everything’. With these final themes we appeared to have achieved our aim of exploring the clinicians’ experiences of using and implementing the QUiPP app. Verbatim quotes have been included within the themes, using the participant numbers seen in Table 1.

## Theme one: experience of using the app

### An accessible and acceptable tool

Clinicians appreciated that the QUiPP app was accessible, and its availability on a smart phone was perceived positively in terms of being quick and easy to use, especially in the busy acute hospital setting. “…it’s hard to get on the computers. So the fact that we can use our phones means that you don’t get slowed down.” [P16]. Clinicians also liked that the app was “easy to access” [P13] with a simple and straight-forward user interface. Such features were felt to help integrate QUiPP’s use into individual practice with ease. “From the moment I downloaded the app and I used for the first time I kept using for every single patient.” [P18]. Acceptability was perceived to be higher among younger users: “It was very easy to do because most of the juniors are very happy downloading anything that’s going to happen onto their phone” [P11] with a junior doctor commenting how “I use my smart phone for everything at work so I just think it’s easy” [P14].

However, initial resistance to the innovation was noted. “…some people are a bit funny about ‘oh I don’t want to download it onto my phone’. I think once they then did it, they saw that it wasn’t an issue and it was fine.” [P1]. One senior clinician highlighted that the correct use of the app may be impaired by the experience of the user. Some junior clinicians may not fully understand or misinterpret the obstetric definitions in the app fields (for example, cervical surgery does not include punch biopsy, and previous preterm birth does not include iatrogenic deliveries [e.g. if a woman was induced at 32 weeks for pre-eclampsia]): “…it’s potentially misinterpretation…based on the inexperience of the doctors.” [P6].

### App threshold felt right

The 5% risk of delivery within seven days was intended as guidance only, but it was important to explore how this was perceived by our clinician stakeholders in actual use. Clinicians agreed that they liked using the 5% threshold as a guide to decision-making, with one research midwife responsible for training clinicians in using the app saying: “I think the 5% is quite comfortable.” [P3].

Clinicians involved in direct clinical care also expressed trust and belief in the risk scores given by the app, with one participant noting how there is: “… a big trust in the app [P10]”, and another participant feeling “I think that people…did trust’’ the app [P9].

### Supportive tool not a clinical crutch

Understanding the QUiPP App as a supportive clinical tool, rather than a dogmatic diagnostic tool proved challenging. When explaining the app to colleagues, clinicians had to: “really push them to see past that 5% and actually see that it was just a guidance” [P1], reminding them that the QUiPP app: “…is a tool and it shouldn’t override your clinical judgement” [P18]. Examples were provided of clinicians using their judgement alongside the app to provide woman-centred care: “…a couple of times I have admitted them…despite the risk being lower than 5% …probably only in 2 cases, very anxious patients which usually comes together with a very bad history and anxiety.” [P18]. The interviewee’s use of ‘despite’ in the phrase “despite the risk being less than 5%” suggests a lack of awareness that this use reflects the intended QUiPP use, rather than a breach of use.

## Theme two: how QUiPP risk changes practice

### Reconsidering risk

Clinicians found that the QUiPP app risk scores made them reconsider their perception of risk. For most clinicians, the scores adjusted their perceptions of risk to a lower level: “…most women actually have a very low risk of going into labour early compared to just saying ‘positive fibronectin’.” [P3].

The percentage risk scores prevented them from categorising the woman into a binary outcome (to admit or discharge). This compelled them to be more analytical in their assessment and management plan:

Previous[ly]…you wouldn’t particularly think whether the patient was likely to deliver or not, you would just think admission or not. Whereas now there is an actual risk, so I think you can personalise the care a bit better. [P3].

Personalised care was also aided by the long-term percentage risks which are also produced by the QUiPP app, in addition to the risk of delivery within one week. This allows for tailored follow-up plans: “…it’s not just about that 5% but it’s about the other percentages as well.” [P1].

This suggests that there is a professional development aspect to using the QUiPP app as it allows clinicians to critically think about preterm labour risk, and how they perceive and manage TPTL.

However, some clinicians thought that utilising the long-term percentage risks “opened up a bit of a can of worms” [P14] in busy acute settings, despite viewing the short-term percentage risks as favourable. The additional long-term information is impractical if clinicians have no pathway that they can offer these women, such as a preterm birth clinic. Meanwhile the short-term risk does not prevent them from admitting or discharging women in the acute setting.

### Confidence in clinical decisions

Not all interviewees felt their risk perception changed but QUiPP’s validation of their existing judgement was still valued: “…it doesn’t really tell you anything you don’t already kind of know in your own head…it’s just trying to put a number on what your own thought process.” [P6].

Presenting the same information in a straight-forward percentage format: “structures their thought process” [P5]. This increased clinicians’ confidence in their clinical decision making as both doctors and midwives felt they had: “an extra layer of prediction into your clinical acumen” [P19].

### “Better conversations” with women

Clinicians found that they had “better conversations” [P6] with women as a result of the QUiPP app. This was because: “women understand percentages” [P16] compared to presenting a fetal fibronectin (fFN) result (often quantitatively in unfamiliar and complicated ng/ml) or a cervical length measurement.

Whilst some clinicians were not aware of the donut infographics (Fig. 1), others found they aided explanations of risk to women:“…I always say to them, you know, 10% risk of delivery before 37 weeks, actually that means that you’ve got a 90% deliver after and that’s where your donuts come in” [P19].

One interviewee also explained how QUiPP’s long-term risk scores aid shared-decision making about returning with further symptoms: “…to the woman…you might say ‘well the risks straight away is quite low so you can go home, but it does sort of suggest you might have a risk so come back if you’re worried’. I mean you always say come back if you’re worried anyway, but I can use that to also help in counselling.” [P11].

Clinicians also highlighted the ability of women being able to download the app themselves; empowering them to be involved in their own health, care plan and decision making: “I like it that the patients can download it themselves… physically seeing the numbers on…their own phone.” [P13].

## Theme three: successfully adopting QUiPP: context is everything

### Time is of the essence

The majority of participants felt the QUiPP app improved the use of their time and reduced the cascade of unnecessary intervention. “It’s a tool that potentially can…reduce our workload…especially if we can reduce the amount of, um, steroids and potentially sliding scale in this use.” [P3].

The app was sometimes seen as “something extra” [P1] and “to start with ‘oh something else we’ve got to do’.” [P2]. However often clinicians thought “it was going to take more time than it actually did” [P12]. Some did not mind even if it did take more time: “I don’t mind taking a bit [more] time if I was giving better care.” [P16].

### Training and retaining clinicians

New clinicians quickly became engaged in using the QUiPP app, especially in sites were the app was already implemented well: “…when we had new staff come into the unit, sort of rotating round … that sort of enthusiasm would rub off on them…and helped them to engage in using it as well.” [P6].

If enthusiasm was lacking with current staff, clinicians found that this changed when new staff started as they brought “a bit of a renewed energy” [P4].

Staffing pressures were noted at nearly all the sites, which affected how successfully the QUiPP app was adopted. For example, clinicians highlighted that frequent utilisation of locums instead of regular staff hindered implementation of the app. Clinicians also discussed how working in a busy, short-staffed unit meant that additional skills (such as cervical length scanning) are not taught: “We get junior doctors and trainees who…are great in triage, but obviously they require training for the cervical length and there is a high turnover…the cervical length training takes a while.”[P3]. If clinicians are not taught how to undertake a cervical length, then they can only ever use the QUiPP app on the qfFN function.

### Conflicting attitudes towards change

Whilst staffing shortages hindered utilisation, more than one site mentioned a lack of cohesion among the consultant body which impaired implementation. Participants also thought that junior clinicians were the most enthusiastic adopters, while some senior clinicians seemed sceptical: “I think sometimes it’s just a general ‘oh, you know, we’ve been doing it this way for a long time so, you know, we don’t necessarily want to try something new’, or … um, ‘I trust my experience more’” [P4].

Participants felt that having positive senior colleagues or consultants who repeatedly endorsed the app helped implementation. Other clinicians felt this endorsement could be from any member of the multi-disciplinary team “there are like these two midwives that [have]…taken that as their responsibility, it’s definitely made a big difference.” [P10]. This demonstrates that if someone was willing to take on ownership and responsibility for the app, they could have a positive impact on the whole multidisciplinary team, despite not being the most senior clinician. In some units midwives expressed the view that using QUiPP was the doctor’s responsibility alone, while other midwives felt it was their responsibility to “encourage them” [P1] to use the app. This may reveal as much about interdisciplinary cohesion as QUiPP itself, but the successful adoption of the app is likely to require engagement and ownership from all stakeholders in preterm labour triage, not just the clinicians who input the clinical data into the app.

The culture towards innovation within their department also had a large effect on implementation of the app. Some units seemed to have a negative attitude towards change: “Honestly people just don’t care…Why would they care? They just want to get through their shift.” [P14]. Other units were more positive: “as a unit I think we are very … are very open to change and development and improvement to patients” [P6]. One site implemented both fFN and the QUiPP app at the same time and reported a very positive attitude to using the QUiPP app. This may have been because at this site, the QUiPP approach to risk did not need to supplant established preconceptions around binary (positive or negative) fFN values.

## Discussion

In this study we interviewed 19 clinicians from 10 hospital sites across three areas in England (London, the South East of England, and the Midlands area of England). This is the first study to explore the experiences of clinicians using a predictive tool to support clinical decision making in care of women with TPTL. The findings also provided insights into how the QUiPP app was implemented in different hospital sites.

Limitations of this study include how participants were not given the opportunity to review the transcripts or findings. Some hospitals had higher workloads than others, meaning clinicians were unable to be interviewed, leading to representation from only 10 of the 13 sites involved in the EQUIPTT study. However, we feel that we achieved representation from a diverse range of sites with varying birth rates, a mix of district general and tertiary units and a range of clinical backgrounds. While participants were not part of the co-ordinating research team or involved in the development of the app, some clinicians interviewed were involved in overseeing research at their local hospital site.

Clinicians appear to find the QUiPP app an accessible and acceptable clinical tool in the triage process, that QUiPP app scores changed how they perceived TPTL risk and increased their confidence in clinical decision making. Clinicians also felt the QUiPP scores allowed them to have better conversations with women they cared for. Given what is known about the uncertainty and conflict women experience during TPTL and how interactions with professionals impact this stressful experience [28], these findings support the use of QUiPP in this setting.

While most clinicians felt the QUiPP app gave them more confidence in their clinical decision making and enhanced communication with women, some participants found it challenging to view the QUiPP app as a supportive but non-dogmatic tool. Concerns have been raised that technology triage tools can prevent holistically assessment [15], which highlights the importance of educating and reiterating that the QUiPP app is a tool to *support* clinical decision making when implementing into hospital sites.

Some participants felt that the app may be used incorrectly due to misinterpreting the obstetric definitions in the app fields. Whilst an information section is available within the app which contains a detailed guide to the field definitions, this was clearly not always utilised. Therefore, future versions of the app will ensure that the user guide is more accessible and/or the app fields are clearer. Other feedback included requests for determining whether, with more data, there are additional risk factors that could be included in the app, as well as including confidence intervals when the app produces a result. Whether adjustments are made to the app itself, or in how the app is implemented at sites, requires further study.

These interviews revealed that some sites did not utilise the long-term preterm birth risks that the app provides. This may be because they do not currently have a mechanism to care for these women who have a low short-term risk, but a higher long-term risk. As the recently published Saving Babies Lives Care Bundle Version 2 [29] is applied across England, it is hoped that preterm birth clinic provision will improve and the pathway for women more established.

The ease of QUiPP app implementation varied between hospital sites. Our interviews did not suggest that these difference in implementation were associated with type of hospital unit or clinician qualification. Yet staff shortages, busy acute settings and hospital culture all seemed to affect the degree of success. However, once clinicians started using the app, the majority felt it improved the use of their time. As service providers strive to encourage a workplace culture which is open to change, learning and improvement, future implementation efforts for QUiPP will need to reconsider how to deliver training to this dynamic workforce and embed this innovation within existing practices.

The increasing need for a preterm birth clinical decision tool for clinicians has been recognized [2]. This study demonstrates that the majority of clinicians find the QUiPP app accessible and helpful in clinical decision making [14]. However, our findings suggest a need to highlight that QUiPP is a clinical decision support tool, rather than a diagnostic tool, and that the app field definitions may need clarification. These are two important areas that require emphasis when implementing the app in new hospital sites.

## Conclusions

This study explored different clinician’s experiences of implementing the app in England. The organizational and cultural context at different sites appeared to have a large impact on how well the QUiPP app was implemented. Future work needs to be undertaken to understand how best to embed the intervention within different settings. This will inform scale up of QUiPP app use across the UK and ensure that clinicians have access to this free, easy-to-use tool which can positively aid clinical decision making when caring for women in TPTL.

## Data Availability

The datasets generated and/or analysed during the current study are not publicly available but are available from the corresponding author on reasonable request.

## Abbreviations

TPTL: Threatened preterm labour
qfFN: Quantitative fetal fibronectin
CL: Cervical length
EQUIPTT study: The Evaluation of the QUiPP app for Triage and Transfer
